# Complete Genome Sequences of Gram-Positive Opportunistic Pathogens Isolated from Hospitals in Almaty, Kazakhstan

**DOI:** 10.1128/mra.00093-22

**Published:** 2022-03-14

**Authors:** Ilya S. Korotetskiy, Ardak B. Jumagaziyeva, Bahkytzhan Kerimzhanova, Oleg N. Reva, Sergey V. Shilov, Tatyana Kuznetsova, Ludmila Ivanova, Nadezhda Korotetskaya, Aimana Bekmukhamedova, Ganiya Satylgankyzy, Nailya G. Klivleyeva

**Affiliations:** a Scientific Center for Anti-Infectious Drugs, Almaty, Kazakhstan; b Centre for Bioinformatics and Computational Biology, Department of Biochemistry, Genetics, and Microbiology, University of Pretoria, Pretoria, South Africa; c Department of Vascular Surgery, National Scientific Center of Surgery named after Syzganov, Almaty, Kazakhstan; d The Research and Production Center for Microbiology and Virology, Almaty, Kazakhstan; University of Rochester School of Medicine and Dentistry

## Abstract

The appearance of drug-resistant pathogens reduces the therapeutic applicability of antibiotics and increases the rate of hospital infections among patients. Complete genome sequences of four Gram-positive clinical isolates of Streptococcus and Staphylococcus were obtained and analyzed to serve as model microorganisms for further studies on drug-induced antibiotic resistance reversion.

## ANNOUNCEMENT

Antimicrobial resistance (AMR) is one of the main threats to human health. Monitoring the spread of genetic determinants and keeping under control the etiological and taxonomical composition of nosocomial pathogens will allow timely identification of threats to human health (1).

Streptococcus pneumoniae, Staphylococcus epidermidis, and two strains of Staphylococcus aureus were isolated in 2021 in the Department of Vascular Surgery of Syzganov’s National Scientific Center of Surgery in Almaty, Kazakhstan. Isolates were obtained by plating from biological material on selective and differential-diagnostic media (Table 1). This study was approved by the Committee of Institutional Animal Care and Use of the Scientific Center for Anti-Infectious Drugs (SCAID), Almaty, Kazakhstan (2). For more details on the isolates, see NCBI BioProject number PRJNA754843. For DNA extraction, cultures were grown on nutrient agar (HiMedia) for 24 h at 37°C. DNA was extracted using a PureLink genomic DNA minikit (Invitrogen, USA). DNA was sheared using the Megaruptor shearing kit 3. The DNA library was prepared using a PacBio SMRTbell Express template prep kit 2.0. SMRTbell templates were annealed using a Sequel binding and internal control kit 3.0. The Sequel sequencing kit 3.0 and SMRT cell 1M v3 tray were used for sequencing. For each SMRT cell (Pacific Biosciences), 600-min movies were captured using the PacBio Sequel-I sequencing platform by Macrogen (Seoul, South Korea). Statistical parameters of the generated reads are shown in Table 1. Default settings were used for all software unless otherwise noted. DNA reads were quality controlled and checked for remaining adapters using LongQC 1.2.0c (3). Assembly and circularization of contigs were performed using Canu 2.0 (4). Plasmid contigs were identified using Platon 1.6 (5). Contigs were joined using MeDuSa at http://combo.dbe.unifi.it/medusa (6) using the most similar reference genomes identified in GenBank using BLASTN (Table 1). The consensus sequences were annotated on the RAST server (https://rast.nmpdr.org/) using the RASTtk algorithm (7) with the fix frameshifts setting. Chromosomal sequences were edited with Artemis 14.0.0 (8) to start with *dnaA* on the positive strand, and the plasmid sequences were shifted for 50 kb to check circularization by mapping the initial PacBio reads against the obtained contigs using pbmm2 (SMRT-Link 10.10.119588). The final consensus sequences were generated from the alignments of mapped reads, annotated using GenBank PGAP, and deposited at the NCBI (Table 1). Multilocus sequence typing (MLST) was performed using the Bacterial Isolate Genome Sequence Database (BIGSdb) (https://bigsdb.pasteur.fr/) and Copenhagen Business School (CBS) (www.cbs.dtu.dk/services/MLST) databases (9, 10).

**TABLE 1:** Deposited sequences of complete genomes of Gram-positive isolates^*a*^

Strain name	Sample type	Media	Total reads/*N*_50_ (bp)	Coverage (×)	Replicon	Length (bp)	GC content (%)	Resistance^*b*^	MLST/serotype^*c*^	Reference genome (GenBank accession no.)	GenBank accession no.	SRA no.
Streptococcus pneumoniae SCAID PHRX1-2021	Swab from pharynx	CHROMagar Orientation, Endo agar	149,806/4,594	386	Chromosome	2,098,200	39.72	OX, AMP, E	ST377	LR216047	CP082820	SRR15674694
Staphylococcus epidermidis SCAID OTT1-2021 (597)	Swab from ear	CHROMagar Orientation, Endo agar	209,855/10,195	405	Chromosome	2,099,244	32.35	AMX, E, AMP, AZM	ND	CP028282	CP082816	SRS9980277
				405	Plasmid 1	24,456	27.9			MW364979	CP082817	
				405	Plasmid 2	24,520	29.15			CP030247	CP082818	
				405	Plasmid 3	13,203	28.99			LR735442	CP082819	
Staphylococcus aureus SCAID OTT1-2021 (597/2)	Swab from ear	CHROMagar Orientation, Endo agar	202,151/10,148	804	Chromosome	2,737,085	32.97	AMX, OX, AMP	ST508	CP047795	CP082813	SRS9980279
				804	Plasmid	33,923	28.19			CP029650	CP082814	
Staphylococcus aureus SCAID WND1-2021 (598)	Swab from wound	CHROMagar Orientation, Cetrimide agar	200,444/10,267	654	Chromosome	2,889,511	32.83	AMX, AMP	ST30	CP047791	CP082815	SRS9980278

a The susceptibility was evaluated by the disk diffusion method in Mueller-Hinton agar (HiMedia, India). The results of the threshold inhibition zones were evaluated according to the CLSI.

b AMP, ampicillin; AMX, amoxicillin; Е, erythromycin; OX, oxacillin, intermediate resistance. The resistance to antibiotics was determined experimentally.

c ND, not detected.

The MLST of Staphylococcus epidermidis strain SCAID OTT1-2021 (597) could not be identified because the *apoE* gene variant of this isolate did not match any known variants. S. aureus isolates belong to sequence type 508 (ST508) (strain 597/2) and ST30 (strain 598). The S. pneumoniae strain belongs to ST377.

### Data availability.

The genomes are available from NCBI BioProject number PRJNA754843 under the accession numbers shown in Table 1.
